# Development of a core outcome set for effectiveness studies of breech birth at term (Breech-COS)—an international multi-stakeholder Delphi study: study protocol

**DOI:** 10.1186/s13063-022-06136-9

**Published:** 2022-04-04

**Authors:** Shawn Walker, Tisha Dasgupta, Andrew Shennan, Jane Sandall, Catey Bunce, Phoebe Roberts

**Affiliations:** 1 King’s College London, Department of Women and Children’s Health, Faculty of Life Sciences and Medicine, King’s College London, London, UK; 2 The Royal Marsden Foundation Trust, London, Sutton UK; 3 London, UK

**Keywords:** Consensus, Core outcome set, Delphi, Breech presentation, Clinical trial

## Abstract

**Background:**

Women pregnant with a breech-presenting foetus at term are at increased risk of adverse pregnancy outcomes. The most common intervention used to improve neonatal outcomes is planned delivery by caesarean section. But this is not always possible, and some women prefer to plan a vaginal birth. A number of providers have proposed alternative interventions, such as delivery protocols or specialist teams, but heterogeneity in reported outcomes and their measurements prevents meaningful comparisons. The aim of this paper is to present a protocol for a study to develop a Breech Core Outcome Set (Breech-COS) for studies evaluating the effectiveness of interventions to improve outcomes associated with term breech birth.

**Methods:**

The development of a Breech-COS includes three phases. First, a systematic literature review will be conducted to identify outcomes previously used in effectiveness studies of breech birth at term. A focus group discussion will be conducted with the study’s pre-established Patient and Public Involvement (PPI) group, to enable service user perspectives on the results of the literature review to influence the design of the Delphi survey instrument. Second, an international Delphi survey will be conducted to prioritise outcomes for inclusion in the Breech-COS from the point of view of key stakeholders, including perinatal care providers and families who have experienced a term breech pregnancy. Finally, a consensus meeting will be held with stakeholders to ratify the Breech-COS and disseminate findings for application in future effectiveness studies.

**Discussion:**

The expectation is that the Breech-COS will always be collected in all clinical trials, audits of practice and other forms of observation research that concern breech birth at term, along with other outcomes of interest. This will facilitate comparing, contrasting and combining studies with the ultimate goal of improved maternal and neonatal outcomes.

**Trial registration:**

Core Outcome Measures in Effectiveness Trials (COMET) #1749

## Introduction

### Background and rationale

Women pregnant with a breech-presenting foetus at term are at increased risk of adverse pregnancy outcomes [1]. The most common intervention used to improve neonatal outcomes is planned delivery by caesarean section [2]. But this is not always possible [1, 3], and some women prefer to plan a vaginal birth [4–6]. Studies of women’s preferences with balanced counselling indicate that as many as 30–50% of women would choose to plan a vaginal breech birth, if this option were appropriately supported [7, 8]. A number of providers have proposed alternative interventions, such as delivery protocols or specialist teams, but heterogeneity in reported outcomes and their measurements prevents meaningful comparisons [9]. This project is the first stage in a larger project that seeks to evaluate an alternative model of care, OptiBreech Care (NIHR300582).

Careful selection of primary and secondary outcomes is a crucial component of clinical trial design [10]. Heterogeneity in outcomes measured within the same clinical area, including inconsistency about the broad domains considered, the outcomes themselves, the way these outcomes are labelled and defined and the methods and timing of measurement, can complicate or prevent useful synthesis and meta-analysis [11]. Standardised reporting of outcomes and their measurement enables the direct comparison of effects of different interventions across multiple studies in ways that minimise bias [10]. This improves the quality of effectiveness research and minimises research waste.

Standardising outcomes through a core outcome set for effectiveness studies of breech birth at term (Breech-COS) will reduce heterogeneity in reporting, enable meaningful meta-analysis, facilitate valid comparisons of new management strategies and improve clinical trial quality. The development of core outcome sets is recommended by the Cochrane Collaboration [11], the Core Outcome Measurement in Effectiveness Trials (COMET) initiative [12] and the Core Outcomes in Women’s and Newborn Health (CROWN) initiative [13].

### Objectives

The purpose of this research is to identify by an international multi-stakeholder consensus a core outcome set for effectiveness studies of breech birth at term (Breech-COS), to enable effective future synthesis and meta-analysis.

The primary objective is to identify the minimum Breech-COS that should be reported in future effectiveness studies of breech birth at term.

The secondary objective is to identify how the items in the Breech-COS should be defined and measured.

### Scope of the core outcome set

The Breech-COS is intended as the international standard for randomised and non-randomised effectiveness studies of breech births in term pregnancies.

## Methods

### Eligibility criteria

Participants included within the scope of the Breech-COS include those with:

1. Breech presentation confirmed from 37 weeks, according to the locally agreed method of dating, in all pregnancies who expect to deliver a foetus in breech presentation
2. No absolute contraindication to vaginal birth, requiring delivery by caesarean section

Health interventions included within the scope of this COS include:

1. Planned modes of delivery (e.g. vaginal birth versus caesarean section, selection protocols)
2. Methods of delivery (e.g. management protocols, maternal birth position)
3. Models of care delivery (e.g. breech team care, midwife/physician/obstetrician as lead carer)

Effectiveness studies comparing methods of turning a foetus to a head-down position in the uterus, such as external cephalic version, are outside the scope of this COS, except in cases where their impact on vaginal breech birth is being studied.

### Existing knowledge of outcomes

The COMET and CROWN databases were searched in April 2020 for other published and unpublished COS development projects relevant to this research, under disease category ‘Pregnancy and Childbirth’ and disease name ‘Breech Presentation’. Only one study was registered in this category; this concerned the practice of acupuncture and moxibustion to promote cephalic version [14] and was thus outside the scope of the Breech-COS.

The following maternity care COS studies are likely to be relevant to the Breech-COS:

- Evaluating Maternity Care: A Core Set of Outcome Measures [15]
- The Composite Adverse Obstetrics Outcomes Study (CAOOS), systematic review published [16], due to be completed in May 2020 (delayed) [17]
- Salutogenic Intrapartum Core Outcomes (SIPCO), systematic review [18] and protocol [19] published, due to be completed in September 2020 (delayed)

In addition, two systematic reviews have reported on consistency, variation and quality of outcomes among COS in maternal and newborn care.

- Duffy et al. (2017), core outcome sets in women’s and newborn health [13]
- Slavin et al. (2021), quality of core outcome sets in maternal and newborn health [20, 21]

When a relevant reporting standard (outcome, definition or measurement) has been agreed through previous COS development, this will be identified to panellists in the Delphi study for their consideration for inclusion in the Breech-COS. Relevant items recommended in previous maternity care COS projects, but not included in previous breech studies, will be proposed for inclusion as appropriate. This is to promote consistency with international standards as much as possible, while enabling justified variation where it achieves consensus among experts in this field.

### Study design: overview

**Fig. 1:**
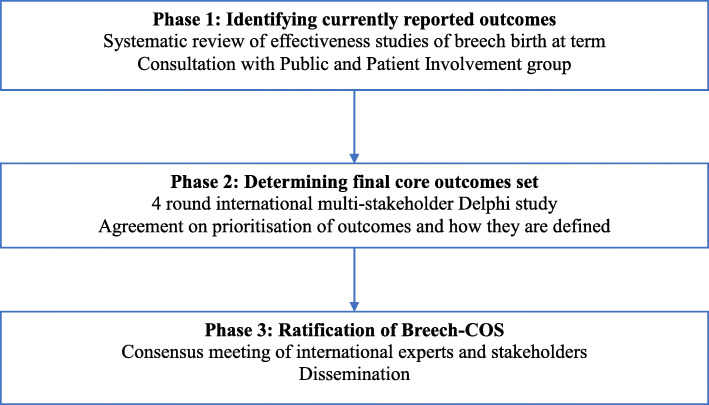
Stages for developing Breech-COS

The design of the study is based on the criteria outlined in the *COMET Handbook* [12]. The Breech-COS will be developed through a three-phase Delphi consensus-building process involving international stakeholders, as shown in Fig 1. Detailed methodology for each phase is provided below.

1. Completion of a systematic review to identify effectiveness and safety outcomes currently reported in effectiveness studies of breech birth at term. An online meeting with the study’s Patient and Public Involvement (PPI) group will be conducted to discuss the importance and relevance of outcomes identified in the systematic review and to ascertain important outcome measures that were not identified through reviewing the literature
2. A four-round Delphi survey, including:
  - Prioritisation of outcomes and generation of a consensus outcomes list in the first two rounds
  - Generation of a consensus on the way these outcomes are labelled and defined, and the methods and timing of measurement, in a further two rounds
3. Ratification of the Breech-COS, including definitions and measurements, in a consensus meeting of international experts and stakeholders

### Study registration

The study has been registered with the COMET initiative (www.comet-initiative.org; # 1749) and has been submitted to the Core Outcomes in Women’s and Newborn Health (CROWN) Initiative. The systematic review will be conducted in accordance with the guidance set out by the PRISMA Statement for reporting systematic reviews.

#### Phase 1: systematic literature review

The purpose of phase 1 is to ensure that an initial list of items for the Breech-COS is grounded in the literature, representing the views of previous researchers [19]. A literature review will be conducted to identify outcomes, definitions and measurements previously reported in effectiveness studies of breech births at term. The results of phase 1 will be a preliminary list of items to be included in a Breech-COS.

##### Types of studies, participants and interventions

RCTs, non-randomised effectiveness studies and systematic reviews of both (with or without meta-analysis) will be included. Studies not describing breech birth outcomes, conference proceedings/abstracts without complete trial description or studies for which full text is not available in English will not be included. Study participants will include all childbearing people ≥37 weeks pregnant with a foetus in breech presentation. Interventions will include:

- Planned modes of delivery (e.g. planned vaginal breech birth versus planned caesarean section, antenatal candidate selection protocols)
- Methods or management plans for vaginal breech delivery (e.g. upright versus supine delivery, expediated versus conservative approached, comparisons of assisted delivery protocols)
- Models of care delivery (e.g. comparisons of care delivered by different experience levels or professions, use of breech teams)

Other interventions intended to modify the outcomes associated with term breech birth may be identified in the review, and these will be included.

##### Search strategy for identification of studies and study eligibility

Full terms of a comprehensive, electronic search strategy are detailed in our published review. MEDLINE, EMBASE and the Cochrane Central Register of Controlled Trials (CENTRAL) databases will be searched. Although searching further databases may have uncovered more literature, it was considered unlikely that the few additional studies indexed would use outcomes unique from those identified in these three databases. The reference lists of eligible studies and reviews will be checked for additional effectiveness studies not identified from the electronic database search. Using Covidence systematic review software, two out of four review authors (Shawn Walker-SW, Alexandra Halliday-AH, Anke Reitter-AR, Tisha Dasgupta-TD) will independently screen the abstracts returned from the search strategy and any studies not meeting inclusion criteria will be excluded. Where necessary to resolve a disagreement, the third member of the research team will be consulted.

Eligibility criteria will include:

- Settings: high- and low-income settings
- Language: all
- Date: 2000 to present
- Study design: systematic reviews, randomised trials, comparative observational studies

This review is designed to identify the choice and consistency of outcomes reported in comparative studies concerning vaginal breech birth. The year 2000 was chosen because this was the year of publication of the single large randomised trial of term breech birth [22]. Two other small trials completed before 2000 are included in the Cochrane Review last updated in 2015 [2]. Our scoping search revealed that no further trials have been completed. Limiting our search of other literature to the last 20 years enables us to survey a wide range of outcomes considered important in smaller comparative observational studies within a reasonable timeframe.

##### Assessment of methodological quality

As the primary objective of this review concerns choice and consistency of outcomes, the overall methodological quality of the included studies from systematic reviews will not be evaluated. Instead, the methodological quality of the reported outcomes in included studies will be assessed using four questions: [12, 23]

1. Is the primary outcome clearly stated?
2. Is the primary outcome clearly defined so that another researcher would be able to reproduce its measurement (e.g. measurement tools, measurement timing)?
3. Are secondary outcomes clearly stated?
4. Are secondary outcomes clearly defined?

##### Data management, extraction, analysis and presentation

Records will be managed using Covidence systematic review software [24]. Independent data extraction will be performed for each article by two review authors using a Microsoft Excel spreadsheet-based extraction form for the following: author and title details, year and journal of publication, study type, setting, key eligibility criteria, study population size, description of intervention, proposed outcomes, primary and secondary effectiveness and safety outcome(s) reported, outcome definition(s), outcome measurement tool(s) and timing(s). Disagreement will be resolved through discussion, involving the third reviewer if helpful. Original study authors will be contacted if there is unclear/unavailable data.

Interventions will be reported based on the Effective Practice and Organisation of Care (EPOC) framework [25]. Reported outcomes will be based on the outcome framework by the Core Outcome Measures in Effectiveness Trials (COMET) and according to the hierarchy as used in the studies (primary, secondary and other). These outcomes will then be condensed into a list for consideration in the Delphi survey.

##### Patient and public involvement on identified outcome measures

As identified by the systematic literature review, the most frequently reported outcome measures will be brought to the study’s PPI group in an online meeting led by members of the team [SW and TD]. The purpose of this stage is to determine [1] how patients and the public with experience of breech birth value the outcomes identified in the published literature: their importance and relevance and [2] to isolate any outcome measures that are deemed important by the PPI group but were not identified by the systematic review. The findings of the discussion will be collated and used to inform the design of the Delphi survey.

(The results of this systematic review were published in 2021 [26], after submission of this protocol for review.)

#### Phase 2: Delphi survey on Breech-COS

The purpose of phase 2 is to ensure the Breech-COS represents international, multi-stakeholder perspectives on the most important outcomes of care for breech births at term. This will [1] ensure it is useful for a range of settings and purposes and [2] increase the likelihood of its use and impact on the quality of future meta-analyses.

Phase 2 will involve a Delphi survey, administered using Qualtrics XM Online Surveys software. In the Delphi method, a consensus is developed anonymously and collaboratively through a series of surveys. Delphi methods have been widely used to develop consensus about COSs and standards of practice [27, 28] in maternity care. The online Delphi will enable the involvement of a wide range of international stakeholders, however limited by Internet access and the ability to speak English.

Participants will be asked to rate the importance of the outcomes identified in phase 1 for inclusion in a Breech-COS and suggest changes if indicated. After each round, the group responses are provided to panellists who can then reconsider their position in light of other viewpoints. Results will be stratified by participant type (e.g. professionals, service users). The anonymity of the Delphi method avoids the opinions of prominent personalities from dominating the consensus and also facilitates wide international participation. All online survey forms will be tested prior to distribution to ensure clarity. This Delphi will consist of two stages, each including two rounds of online surveys, response and feedback. These are further described below.

The results of phase 2 will be a ranked list of the most important outcomes to include in the Breech-COS, including how they should be defined and measured, and identification of any items requiring further discussion.

##### Selection of panel members

The panel in phase 2 will include a minimum target of 50 participants [29]. We aim to recruit a diverse respondent pool, with involvement from each major stakeholder group. These include obstetricians, midwives, neonatologists, researchers, service users, representatives from support groups, service managers, healthcare commissioners, health economists and statisticians, and an option to write-in one’s speciality where this was not prospectively listed. Selected participants will reflect a broad range of clinical experiences and geographical expertise, with representation from high-, middle- and lower-income countries.

Clinicians who have published research concerning the management of breech presentation at term since 2000 will be purposively invited to participate. They will be identified by way of the systematic review being conducted in the first stage. An invitation email will be sent to all identified panel members using a survey, which has been piloted with a subsample of the first round respondents. Authors of clinical effectiveness studies, other forms of comparative effectiveness research, systematic reviews and national guidelines will be preferentially invited to participate. All of those purposely invited will be given a link to forward to colleagues or service users whom they feel should also participate. Similar recruitment strategies have been used in previous COS development [12, 30].

Service users will be eligible for inclusion in the Delphi survey if they have or their partner have experienced a breech pregnancy at term within the past 5 years, and they have a fluent understanding of written English. Representatives from service user advocacy groups will be eligible if they have experience of supporting women and others who have planned breech deliveries. Members of a UK-based PPI group who have already contributed to this project at the funding application stage will be invited to participate. International participation will be sought through established collaborative partnerships with service user groups in North America, Europe and the Antipodes. All service users will have the opportunity to receive training to enable them to understand the purpose of the research and participate fully.

All potential participants will be emailed an invitation letter outlining the aims and details of the study and the rationale and importance of completing the entire Delphi process. Respondents who agree to take part will be assigned a unique identification number. For each round of the process, participants will have 3 weeks to complete the survey with generic email reminders sent at the 1- and 2-week marks. All data will be stored against the unique identifier only; participants will be blinded to the other respondents in the study. Only two members of the research team (SW and TD) will have access to the complete list of Delphi survey panellists. For each round of the Delphi survey, response and attrition rates will be calculated.

##### Delphi round 1

In the first round, participants will be asked to identify the stakeholder group and geographic area to which they belong, and complete questions about their professional background and experience with clinical research relevant to the management of breech presentation at term. They will then be presented with a complete list of effectiveness and safety outcomes generated from the literature review. Outcome order will be randomly assigned to mitigate the influence of display order on scoring. Participants will be asked to rank each outcome on a scale from 1 to 9, based on the Grading of Recommendations Assessment, Development and Evaluation work group definitions [31]. Scores of 1–3 indicate an outcome is not that important for inclusion, scores of 4–6 indicate an outcome is important but not critical for inclusion and scores of 7–9 indicate an outcome felt critical for inclusion in the COS. An option to select ‘Unsure of significance’ will also be available. Participants will be asked to focus on ranking the most important outcomes for inclusion highly and excluding outcomes felt to be of lesser importance; regardless of score, all outcomes will be carried to the second round. Finally, through the free-text entry, participants will have the option to clarify compelling arguments for and against inclusion of outcomes and to identify additional outcomes not included in the first-round questionnaire. Where the results of the SR indicated disparity in definitions or measurement, participants will also be invited to recommend how items should be labelled and defined, and the methods and timing of measurement.

Responses from round 1 will be analysed and collated into a feedback report. Descriptive statistics will be used to summarise the number of participants scoring each outcome and the distribution of scores. Ranking will be determined by the median rating of each outcome. Responses to open-ended questions will be reviewed by the authorship team to evaluate for insights that should be shared with the panel in round 2. Additional suggestions will be reviewed for outcomes not captured or misrepresented in the first-round questionnaire. If participant numbers are large enough to enable meaningful subgroup analysis, scores will be stratified by stakeholder group to evaluate for differences from other panellist responses. Panellists who do not complete the first-round survey will not be invited to participate in round 2.

##### Delphi round 2

In round 2, each participant will be provided with the number of respondents and distribution of scores for each outcome from the first round, stratified by stakeholder group. They will then be shown their own score from round 1 and asked to rescore each outcome, and any additional outcomes arising from round 1, with consideration based on insights from the group. Each outcome will be rescored on a scale from 1 to 9 as previously described. Changes in score from round-to-round will be documented.

Responses from round 2 will be analysed with descriptive statistics. Outcomes that ≥ 70% of panellists scored 7–9 and < 15% scored 1–3 will be decided a priori to have met consensus for inclusion. Conversely, outcomes ≥ 70% of panellists scored 1–3 and < 15% scored 7–9 will be decided to have met consensus for exclusion. Rank order will be determined using the mean score of all responses. Outcomes not meeting these definitions will be classified as lack of consensus. While these definitions are subjective, they have been recommended by previous COS authors and avoid post hoc definitions of consensus that may bias the results [12, 23].

##### Delphi rounds 3 and 4

If necessary, rounds 3 and 4 of the Delphi survey will replicate the methods of the first two rounds in order to achieve a consensus on the way the items in the Breech-COS are labelled and defined, and the methods and timing of measurement [12].

#### Phase 3: consensus meeting to ratify results

The purpose of phase 3 is to enable real-time debate of the results obtained from phase 2 and agree with the final Breech-COS and secondary outcomes. A consensus meeting with key stakeholders will be held in central London after completion of the Delphi process. On-line meeting software will be used to enable participants less able to travel to participate. The meeting will be chaired by Professor Andrew Shennan with the objective of finalising the outcomes for inclusion in the COS. Approximately 30 participants will be purposively sampled from panellists completing all four rounds of the Delphi study, with the intention of ensuring each stakeholder group and broad geographic and economic areas are represented. The results from each round of the Delphi survey will be reviewed and participants will ratify the outcomes that meet consensus criteria for inclusion and exclusion. Participants will then discuss the outcomes which did not meet the criteria for agreement. Based on the discussion, participants will then anonymously vote for each outcome for inclusion and exclusion in the finalised COS using a format similar to that of the Delphi survey. All participants in the final consensus meeting will be acknowledged in the publication of results.

##### Dissemination

Results of this Breech-COS project will be reported according to the COS-STAR criteria [32] and submitted for publication in a leading obstetric or medical journal. They may also be disseminated at appropriate conferences. The expectation is that the Breech-COS will always be collected in all clinical trials, audits of practice and other forms of research that involve breech birth at term. This will facilitate comparing, contrasting and combining studies with the ultimate goal of improved maternal and neonatal outcomes.

## Study status

The systematic review stage of this study was started in June 2020. A meeting with the study’s PPI group was conducted on December 10, 2020, to review the outcomes and this protocol, and the review was published in 2021 [26]. The protocol was first submitted for publication in February 2021. Stakeholder recruitment and round 1 of the Delphi process began with circulation of the round 1 questionnaire in November 2021, which closed in January 2022. We expect completion by December 2022, including the consensus meeting. Following this, the ratified Breech-COS will be reported via publication in an academic journal. This timeline is subject to some flexibility to account for the needs of front-line clinician participants to prioritise clinical care during the COVID-19 pandemic, and uncertainties arising from this.

This is the revised version of the protocol, incorporating peer-reviewer feedback and dated 01/02/2022.

## Data Availability

Data from this study will be completely anonymised and retained on the King’s College London secure infrastructure system for a period of 7 years after the completion of the study. Details of how it can be accessed will be included in the published report, as per NIHR requirements, and the anonymised data set will be made available to other researchers with appropriate ethical clearance upon request.

## Abbreviations

Breech: ‘Breech’ refers to a breech-presenting foetus, lying in a longitudinal position, with buttocks, feet or knees closest to the cervical os (bottom of the uterus)
Breech-COS: A core outcome set for effectiveness studies of breech birth at term
COMET: The Core Outcome Measures in Effectiveness Trials initiative maintains a database of all core outcome set development projects
COS: Core outcome set
CROWN: CoRe Outcomes in Women’s and Newborn Health
NIHR: National Institute for Health Research
Non-randomised effectiveness study: Studies which use a control to assess the effect of an intervention but do not use a random method of allocating participant groups. Examples include non-randomised controlled trials, controlled before-and-after study, interrupted time series study, historically controlled study, cohort study, case-control study and cross-sectional study
PPI: Patient and public involvement
RCT: Randomised controlled trial
SR: Systematic review
Term: ‘Term’ refers to a term pregnancy, defined as a gestation greater than 36 weeks 5 days and less than 42 weeks 0 days
